# The use of typing methods and infection prevention measures to control a bullous impetigo outbreak on a neonatal ward

**DOI:** 10.1186/2047-2994-1-37

**Published:** 2012-11-20

**Authors:** Maike Koningstein, Leon Groen, Kathelijn Geraats-Peters, Suzanne Lutgens, Ariene Rietveld, Petr Jira, Jan Kluytmans, Sabine C de Greeff, Mirjam Hermans, Peter M Schneeberger

**Affiliations:** 1 Department of Epidemiology, National Institute of Public Health and the Environment (RIVM), Bilthoven, The Netherlands; 2 Department of hospital hygiene, Jeroen Bosch Hospital, ‘s-Hertogenbosch, The Netherlands; 3 Department of Microbiology and Infection Control, Amphia Hospital, Breda, The Netherlands; 4 Municipal Health Service ‘Hart voor Brabant’, ‘s Hertogenbosch, The Netherlands; 5 Department of Paediatrics, Jeroen Bosch Hospital, ‘s Hertogenbosch, The Netherlands; 6 Department of Microbiology, Jeroen Bosch Hospital, ‘s Hertogenbosch, The Netherlands

**Keywords:** MSSA, EEFIC, Raman, Bullous impetigo, Neonate

## Abstract

**Background:**

We describe an outbreak of Bullous Impetigo (BI), caused by a (methicillin susceptible, fusidic acid resistant) *Staphylococcus aureus* (SA) strain, *spa-*type t408, at the neonatal and gynaecology ward of the Jeroen Bosch hospital in the Netherlands, from March-November 2011.

**Methods:**

We performed an outbreak investigation with revision of the hygienic protocols, MSSA colonization surveillance and environmental sampling for MSSA including detailed typing of *SA* isolates. *Spa* typing was performed to discriminate between the SA isolates. In addition, Raman-typing was performed on all t408 isolates.

**Results:**

Nineteen cases of BI were confirmed by SA positive cultures. A cluster of nine neonates and three health care workers (HCW) with *SA* t408 was detected. These strains were MecA^-^, PVL^-^, Exfoliative Toxin (ET)A^-^, ETB^+^, ETAD^-^, fusidic acid-resistant and methicillin susceptible. Eight out of nine neonates and two out of three HCW t408 strains yielded a similar Raman type. Positive t408 HCW were treated and infection control procedures were reinforced. These measures stopped the outbreak.

**Conclusions:**

We conclude that treatment of patients and HCW carrying a predominant SA t408, and re-implementing and emphasising hygienic measures were effective to control the outbreak of SA t408 among neonates.

## Background

Bullous Impetigo (BI) is a superficial bacterial skin infection caused by *Staphylococcus aureus* (SA), mainly affecting infants and young children. Bullous impetigo, like regular impetigo, is not a notifiable disease in the Netherlands; therefore there are no data on incidence rates. In the Netherlands, impetigo is the third most common skin disorder in children presented to the general practitioner [1]. In 1987, the incidence rate of impetigo of children under 18 years reported by general practitioners (GP’s) in the Netherlands was 16.5 per 1000 patients, and rose to 20.6 per 1000 patients in 2001[2]. The blistering of the skin in BI is caused by the exfoliate exotoxins ET-A or ET-B [3]. Sometimes BI evolves into the more serious staphylococcal scalded skin syndrome (SSSS) [4-6]. The mortality rate of SSSS in children is less than 5% [6].

An Epidemic European Fusidic Acid-resistant Impetigo Clone (EEFIC) has been described in the United Kingdom, Ireland, France and in Scandinavian countries [7-10]. This clonal SA strain is characterized as *spa* type 171, or single locus variants t408, t659, t874, and t875. The presence of exfoliative toxin A (ET-A) and often ET-B, and low level fusidic acid resistance due to the presence of chromosomal fusB are strongly associated with impetigo [8,9].

Starting in March 2011, neonates presented with a blistering skin disease at the hospital. In May, the hospital infection prevention ward was notified by a resident in paediatrics.

A subsequent investigation was started by the hospital infection prevention ward.

The goals of this study were to investigate possible routes of transmission through observational studies and typing of MSSA, and to establish adequate infection control to stop the spread of MSSA t408 in the hospital.

## Materials and methods

### The hospital

The Jeroen Bosch Hospital is a tertiary teaching hospital with a coverage area of 350,000 of the Dutch population. The number of clinical bed-days in 2010 was 195,269. On average, patients of the gynaecology and obstetrics departments stayed for 2.8 days. In 2010, a total of 2500 in-house deliveries were performed, of which approximately 20% were caesarean sections. The hospital has 45 nursery beds and 4.2 full-time equivalent infection prevention staff. In the end of April 2011 (during the outbreak) the hospital changed location to a newly build hospital in the same city.

### Case definition

Cases were defined as neonates presenting in the hospital with a blistering skin infection (impetigo) with a culture proven SA, starting from 15 March 2011, and notified to the infection control department. A probable case was defined as a neonate with a blistering skin disease, seen by one of the doctors at the Jeroen Bosch Hospital, in the same period of time as a case, but without any laboratory confirmation.

### Cases

In March 2011, a first and second probable case presented at the hospital: both neonates presented with a blistering skin disease. In April, two more probable cases presented with the same blistering symptoms. The first three cases were not hospitalised at the same time. In May, nine more cases presented with IB. These cases had an overlapping hospital stay and were considered a cluster. By this time, the hospital infection prevention department was notified by a resident in paediatrics.

The microbiology and hospital infection prevention department was notified and an investigation was started. Since the beginning of May, all *S. aureus* samples collected from neonates were kept for elaborate typing. In the next three months, another 15 neonates were identified, and after a stretch of two months in which no new cases presented, we found one last case in November. The nursery is a closed unit for in-house births. Infection control nurses performed audits and interviews on adherence to the hygienic and cleaning protocols in the operating room (3×), at the departments of neonatology (2×), and obstetrics (7×). Breaches of Intensive Care practices are corrected in collaboration with HCW from the unit.

### Laboratory methods

#### Spa typing

PCR amplification of the *spa* gene was done as described before [11]. The size of the PCR products was determined on an Agilent 2100 Bioanalyzer (Agilent Technologies Netherlands BV, Amstelveen, The Netherlands) with a sizing accuracy of 10% (http://medsci.udel.edu/cores/bcl/bioanalyzer.html). For the neonatal isolates all *spa* gene PCR products were sequenced. For the HCW *spa* gene, PCR products within the 10% range of the size of *spa* type t408 (415 bp), 415 ± 10% (374–457 bp), were sequenced. For sequencing PCR products were purified by means of USB® ExoSAP-IT® PCR Product Cleanup (Affymetrix UK Ltd). Sequences were generated using the BigDye terminator v1.1 Cycle Sequencing kit (Applied Biosystems/Life Technologies) according to manufacturer’s instructions with the above primers, and analysed using Sequence Scanner software version 1.0 (Applied Biosystems/Life Technologies). The software Ridom StaphType (http://www.spaserver.ridom.de/) was used for *spa* sequence analysis.

### Raman typing

Raman spectroscopy was performed according to the manufacturer’s protocol (SpectracellRA bacterial strain analyzer, River Diagnostics, Rotterdam, The Netherlands). In short, SA strains were analysed after 20-hrs incubation on Trypticase Soy Agar. The biomass was re-suspended in 5μl sterile milliQ and transferred onto the sample carrier. The sample carrier was measured in the SpectracellRA at 785nm laser. The ‘Raman-shift’, indicated as the alteration in wavelength of a laser due energy absorbance of molecules within the bacteria, creates a unique spectrum for each bacterial strain. These spectra of the strains were compared. From the resulting 2-dimensional checkerboard plot clonal relationships were established using the squared Pearson correlation coefficient (R^2^). Instrument settings were set at sensitivity of 92.2%, based on a false positive result of 1.0% and a false negative result of 3.0% as defined by the manufacturer.

### Screening health care workers

All HCW of the neonatal ward and all paediatricians have been requested to participate in screening rounds three times; in May, June and August. To maximize the yield for each person a nose-swab from both the left and right nostril were taken. A total of 564 samples were taken form 226 HCW.

### Outbreak investigation

To analyse whether the t408 strain had been circulating in the hospital before we noticed, a trend analysis was performed on data from January 2007 - September 2011, on all *SA* cases in neonates that occurred within a month after birth. We analysed the amount of FusR of S. aureus (vitek, bioMérieux, Durham, USA) of previous years and compared it to the number of FusR during the outbreak period, using chi-square statistics of SAS 9.2 for Windows (SAS Institute).

As part of a “search and destroy policy” contact screening was performed for each case, meaning that hospital staff, and other hospital patients that were in contact with a case were screened for SA, which is standard practice in case of an MRSA patient, even though this outbreak was due to an MSSA. Only neonates with symptoms were cultured, clinicians were advised to culture neonates with symptoms. As a proxy for a case one sister of a potential case was tested.

In addition to the staff-screening for SA, we also performed environmental cultures using dry cotton swabs from incubators, resuscitation tables, a resuscitation case, trolleys, and various utensils in patient rooms and the OR in May and June.

The communication department and the regional public health department were involved in writing explanatory letters to parents of cases and probable cases.

## Results

### Cases

**Figure 1:**
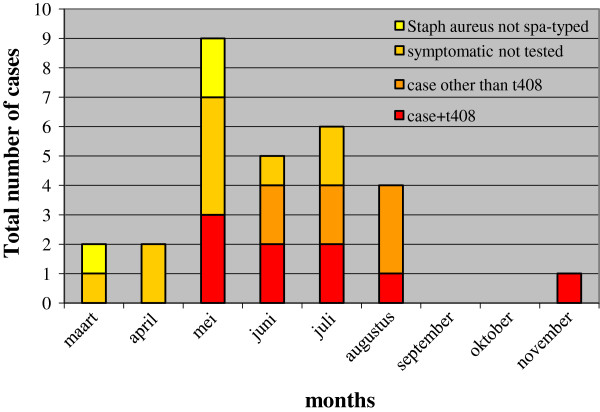
Number of total cases in the Impetigo Bullosa outbreak, Jeroen Bosch Hospital, 's Hertogenbosch, The Netherlands, March-November 2011.

Following the case definition, a total of 19 cases were found. In one case (D) a sister was found positive. Another ten probable cases with clinical impetigo bullosa, but without laboratory confirmation (*spa* typing), were identified, Figure 1. For 26 of the 29 cases and probable cases swabs were obtained yielding SA positive cultures, Figure 1. To discriminate among SA isolates, 16 cultures were available for *spa* typing and yielded various *spa* types with one cluster of 9 of *spa* type t408, Table 1. *Spa* types of the other seven IB patients were t026 (L and M), t279 (E and K), t505 (G), t091 (J) and t9259 (N).

**Table 1:** Overview patients and their spa- and Raman types

**Patient**	**Month**	***Spa*-type**	**Raman**	**Symptoms**
A	May	t408	Separate type	yes
B	May	t408	Cluster	yes
C	May	t408	Cluster	yes
D	June	t408	Cluster	yes
E	June	t279	-	yes (after 11 days)
F	June	t408	Cluster	unknown
G	June	t505	-	yes
				yes, and twin brother
H	July	t408	Cluster	of F
I	July	t408	Cluster	yes
J	July	t091	-	yes
K	July	t279	-	yes
L	August	t026	-	yes
M	August	t026	-	yes
N	August	t9259	-	yes
O	August	t408	Cluster	yes
P	November	t408	Cluster	yes
Staff				
Q		t408	Separate family cluster	no
R		t408	Cluster	non
S		t408	Cluster	no

### Screening of health care workers

In three separate screening rounds a total of 564 samples were taken from 226 HCW, in time-frames of May-June, June-July, and August-October. These 226 HCW represent 96% of the total number of HCW present at the departments during these three screening rounds. A total of 131 SA positive cultures (23%) were found. Three isolates (0.5%) were identified as SA *spa* type t408. The HCW carrying t408 were a medical officer-paediatrician (R, nasal carriage) and two nurses (Q and S, both with nasal and throat carriage). One of the isolates (from Q) contained both *spa* type t408 and t171, a single locus variant of t408. Positive staff members were restricted from the hospital for five days during the treatment period and were treated with a combination of Bactroban skin and nose ointment (for nose and perineum), Chloor hexidine suction tablets, Hibi scrub shampoo, oral Trimethoprim bd 200 mg, and Rifampicine od 600 mg for 10 days. All HCW ceased to be carriers after treatment.

### Further characterization of spa type t408 positive SA isolates

All 12 identified SA t408 strains were fusidic acid resistant (EUCAST criteria) with MIC values 3–8 mg/L, while the non-t408 *SA* strains were fusidic acid sensitive. In addition, the *SA* t408 strains were MecA^-^, PVL^-^, ETA^-^, ETB^+^, and enterotoxin A-D negative.

Raman typing of the t408 strains (9 patients and 3 HCW) yielded one large Raman cluster with 8 cases (B-D, F, H, I, O and P) and as well as 2 HCW (R and S). Two other Raman types were found for the isolate of HCW Q and the isolate of neonate A, Table 1.

### Observation of infection control measures

Environmental swabs (n=73) taken from two resuscitation tables as well as from all instruments used for resuscitation of neonates, were negative.

Environmental cultures (n=73) from swabs taken from incubators, two resuscitation tables, a resuscitation case, trolleys and various utensils in patient rooms, and the OR in the months May and June were all negative. Observation and audits on adherence to infection control practices yielded numerous targets for improvement such as disposing of disposables, cleaning procedures, storage, dress code and hand hygiene.

## Discussion

Treatment of healthcare workers carrying SA t408, and re-implementing and re-emphasising hygienic measures were effective to control the outbreak of SA t408 among neonates in the Jeroen Bosch Hospital. Rapid typing tools are indispensable to support adequate infection control measures. No new cases have been reported since November 2011. However, increased awareness is maintained in our setting to identify new cases. Furthermore, it is recommended that all fusidic acid resistant strains isolated from neonates are typed and analysed for possible clustering with other isolates in our hospital.

Routine surveillance of MSSA is not common practice in The Netherlands, so the epidemiology of MSSA and of specific methicillin susceptible isolates, such as t408, is largely unknown. This MSSA type, t408, is possibly linked to the EEFIC clone, which has caused outbreaks at neonatal wards causing BI and SSSS in several European hospitals [8,10,12]. EEFIC is most commonly seen as t171 but has also has been described as single locus variants t408, t659, t874, and t875 [9]. All belong to MLVA complex 123 [pers. comm., de Neeling].

All newly identified *SA* t408 strains were fusidic acid resistant (EUCAST criteria) with MIC values 3–8 mg/L, while the non-t408 SA strains were fusidic acid sensitive. Considering the number of fusidin resistant (R) S aureus isolated from 2007 until 2011 in neonates in our hospital (total of 188 MSSA S aureus isolates on unique patients of max. 31 days old), we noticed a significant increase starting from the third quarter in 2010. In addition, the here described *SA* t408 strains were MecA^-^, PVL^-^, ETA^-^, ETB^+^, and ETAD^-^, like most other EEFIC strains[7-10,13,14].

Recently, Rijnders et al. [15] studied this EEFIC clone in the South of the Netherlands by examining skin and soft tissue infections (SSTI) at GPs in 2007–2008. Of all participating SSTI patients seen by GPs in the southern part of the Netherlands, approximately 2.5% carried t408. This finding leaves room to speculate whether the cases described here were either related to a local outbreak, or a representative sample of the MSSA circulating in the community at the time. On the other hand, re-implementing stricter hygienic protocols did have an impact on the amount of new cases, suggesting a local outbreak was controlled.

Moreover, no overlap in hospital stay between was seen for some of the clustered cases. Patient P (Table 1) was found in November of 2011, three months after the last case of t408 was found. Patient A - the first case to be *spa* typed - had an unrelated Raman-type compared to the cases that were typed later.

Unfortunately, we have no data on frequencies of circulating methicillin susceptible SA *spa* types in the area of our hospital. This study shows *spa* type t408 in nine out of sixteen neonatal cases, in contrast to surveillance cultures among HCW which yielded only three t408 among 131 isolated MSSA. It is possible that t408 is the predominant strain amongst neonates in our area.

Raman typing appears to be a convenient method for epidemiological typing in an outbreak situation. It has superior discriminative power compared to *spa*-typing, which was not specific enough to distinguish between patient A (Table 1) and the cluster that was found. Patient A was the first typed t408 patient and had an unrelated Raman type. Raman-typing is highly specific, is fast (within an hour) with high throughput, and is an easy-to-use method for outbreak situations [16]. In this study both *spa*-typing and Raman typing were used. Ideally, real time results identifying the incriminated strain would be most desirable to adequately install infection control measures. *Spa*-typing is more laborious than Raman typing and has possibly less discriminative power for this typical application (i.e. *S. aureus* t408). Raman typing is an expensive technique and specific equipment is needed. An important drawback of Raman typing is that the output data are not easily comparable between different databases since the output data can not be translated into exact numbers (as in *spa* typing). Therefore, Raman typing is not suitable for large scale outbreak settings or (inter)national surveillance.

From the observation at the operation theatre we concluded that it was highly unlikely that a member of the operation team could contaminate the neonate, since there is no direct skin contact between the operating team and the neonate. However possible sources of contamination existed because the resuscitation table was not properly cleaned, materials from previous caesareans were still present at the table, and a cleaning protocol for the resuscitation table did not exist.

Our advice was to devise a protocol for the cleaning of the table, and to write a protocol for twin deliveries, since this was missing.

During the observation of the neonatal ward we concluded that the resuscitation table, emergency trolley, and the incubators were not cleaned properly. Therefore we took environmental swabs of these surfaces as well. We also concluded that in the storage room no clear division between sterile and non-sterile products was maintained. We also noticed that a clear cleaning protocol was missing. These points were taken to the relevant wards/nurses and the hygienic protocols were sharpened/re-implemented.

Because most of our IB cases were caused by MSSA strains other than t408, we are interested to do a follow-up study where we will not only focus on bullous impetigo, but also on other impetigo cases seen in child day-care centres and GPs, to get a better understanding of the epidemiology of virulent MSSA strains amongst infants and older children.

Our outbreak peaked early summer in May, and lasted from March until August, with one last case in November. It seems unlikely that this outbreak can be linked to the yearly increase in impetigo cases that is seen in day-care centres and nurseries in late summer, since our outbreak didn’t peak in late summer [1,2,17].

## Conclusions

In conclusion, we think this outbreak could have been caused by failing infection control practices and shortcomings in infection control protocols. Another option is that this outbreak is a reflection of bullous impetigo found in the general population outside of the hospital; the EEFIC cluster is regularly seen in European hospitals. We consider Raman typing to be useful for use in outbreak situations in small settings. Finally, we would advocate gathering more surveillance data on circulating methicillin susceptible SA strains.

## Abbreviations

BI: Bullous Impetigo; EEFIC: Epidemic European Fusidic Acid-resistant Impetigo Clone; ET: Exfoliative toxin; GP: General practitioner; HCW: Healthcare worker; MRSA: Methicillin resistant *staphylococcus aureus*; MSSA: Methicillin susceptible *staphylococcus aureus*; SA: *Staphylococcus aureus*; SSSS: Staphylococcal scalded skin syndrome.

## Competing interests

The authors declare that they have no competing interests.

## Authors' contributions

LG is the infection prevention specialist and has been responsible for the audits and the revision of the hygienic protocols. PS is the main supervisor of this article and KGP, MH and PS are responsible for the lab work, analyses and coordination from the Jeroen Bosch Ziekenhuis. SL and JK facilitated the Raman typing and did the analyses of that part. AR contributed to the communication with parents and local health authorities. MK has been responsible for analysing and writing of the manuscript. All authors had a substantial intellectual part in the study and all have contributed to the writing of the manuscript. All authors have read and approved the manuscript.
